# How postgraduate trainees from different health professions experience the learning climate within an operating theater: a mixed-methods study

**DOI:** 10.1186/s12909-019-1648-1

**Published:** 2019-06-21

**Authors:** Kasana Raksamani, Renée E. Stalmeijer

**Affiliations:** 1 Department of Anesthesiology, Faculty of Medicine Siriraj Hospital, Mahidol University, Bangkok, 10700 Thailand; 2 Department of Educational Development and Research, Faculty of Health, Medicine and Life Sciences, Maastricht University, Maastricht, The Netherlands

**Keywords:** Learning climate, Operating theater, Postgraduate trainees, Interprofessional learning, Supervision

## Abstract

**Background:**

The learning climate within a learning environment is a key factor to determine the potential quality of learning. There are different groups of postgraduate trainees who study primarily in the operating theater (OT), which is a complex, high-stake environment. This study created and validated an interprofessional measure of the OT educational climate and explored how postgraduate trainees from different health professions experienced the learning climate within the operating theater.

**Methods:**

An explanatory, sequential mixed-method design was used. The quantitative phase used and validated a newly developed questionnaire, the Operating Theater Educational Climate Test (OTECT), to evaluate the perceptions of anesthesia residents, surgical residents and student registered nurse anesthetists. In the qualitative phase, three mono-professional focus groups participants’ opinions on the factors influencing their learning climate were explored.

**Results:**

The OTECT questionnaire was found to be valid. The questionnaire response rate was 78.9% (142 respondents from 180). Questionnaire results indicated similar perceptions of the OT learning climate by learners from all disciplines. Focus groups revealed three major influencing factors on the experienced learning climate: 1) nature of work in the OT, 2) the role of the supervisor, and 3) the interprofessional dimension of work in the OT.

**Conclusions:**

The OT learning climate was perceived similarly by trainees from three health profession. The high stakes nature of the OT inhibited learning most as it impacted both trainees and supervisors. The results can be applied to improve the overall learning environment in the OT for all groups of learners.

**Electronic supplementary material:**

The online version of this article (10.1186/s12909-019-1648-1) contains supplementary material, which is available to authorized users.

## Background

The learning climate can be defined as the physical and psychological environment including how trainees perceive the overall teaching and learning condition and educational tone [1, 2]. Studies have revealed that a positive learning climate is a key factor for effective learning in different models of clinical, workplace based learning [3, 4]. Furthermore, the learning climate as experienced in postgraduate training programs has been linked to learning and clinical outcomes [5–7] as well as trainees’ long-term practice patterns in terms of quality and safety of patient care [8, 9]. In order to evaluate and improve the learning climate, valid and reliable instruments are needed [10]. Furthermore, the multifaceted nature of the experienced learning climate necessitates the consideration of specific characteristics of each environment [10, 11].

The Operating Theatre (OT) is an important learning setting for many postgraduate trainees in various health professions like surgery, anesthesiology, and nurse anesthesiology. For all these trainees the OT is the main working and learning environment: all have their own learning goals and all are simultaneously trying to benefit from the various learning opportunities [12]. The OT as a learning environment can be considered to be especially complicated as it involves collaboration between multiple disciplines, has a diversity of sensory input, is characterized by time pressure, very high stakes and requires resource intensive work [13, 14]. All these factors combined can result in different perceptions about learning opportunities and barriers for postgraduate trainees present within the OT.

An important factor that might influence the perceived learning climate in the OT, is the interprofessional teamwork that characterizes this environment. Many different professions are involved in taking care of a patient in the OT making interprofessional teamwork essential to warrant patient safety and the patient outcome [15, 16] which is known to cause tensions [17]. Recent studies revealed the increasing importance of informal interprofessional learning in the workplace to improve quality of clinical learning [18, 19]. The postgraduate trainees in the OT consequently need to collaborate with other professions and learn to work as a team.

Measuring the quality of the learning climate creates the opportunity to improve the quality of trainees’ learning by redesigning the workplace curriculum and providing faculty development to improve and tailor the learning environment to student needs [20, 21]. The Dutch Residency Educational Climate Test (D-RECT) is a widely used tool to measure the learning climate as experienced by residents and measures different constructs related to the integration of working and training in the clinical workplace like supervision, collaboration and feedback [22]. Additionally there are two instruments specifically aimed at measuring the OT learning climate, the Surgical Theatre Educational Environment Measure (STEEM) [23] and the Anesthetic Theatre Educational Environment Measure (ATEEM) [24]. While the D-RECT measures the general learning environment, the STEEM and ATEEM specifically pay attention to the importance of interprofessional teamwork and the high-stakes nature of learning in the OT.

Thus far measuring the clinical learning climate has been approached from a mono-professional perspective [11, 22, 24]. However, the same learning climate could be experienced differently from the perspective of the different learners present within the same environment. This warrants an interprofessional measure of the learning climate. Therefor the aim of this study was twofold 1) design an interprofessional measure of the OT learning climate and 2) examine the different perceptions of the learning climate in the operating theater among different learners from different professions as well as identify the factors contributing to the perceived learning climate.

## Methods

### Context & Participants

The Faculty of Medicine, Siriraj Hospital, is a 2200-bed tertiary care center in Bangkok, Thailand, with an undergraduate medical school and postgraduate training centers for many specialties. There are 60 operating theaters, which are major training venues for surgical residents (*N* = 70), anesthesia residents (*N* = 70) and student registered-nurse anesthetists (*N* = 40). The characteristics of each training program are included in Table 1 for the transferability purposes of the qualitative data interpretation. Participants were all training at Siriraj Hospital at the time of this study and were recruited by a research assistant who had no professional relationship with any participants.

**Table 1:** Characteristics of the respondents and the training programs

Demographics		All respondents (*N* = 142)	Surgical residents 59 (41.5%)	Anesthesia residents 56 (39.4%)	Student nurse anesthetists 27 (19%)
Gender	Male	48 (33.8%)	37 (62.7%)	11 (19.6%)	0 (0%)
	Female	94 (66.2%)	22 (37.3%)	45 (80.4%)	27 (100%)
Age	20–25	4 (2.85%)	0 (0%)	2 (3.6%)	2 (7.4%)
	26–30	120 (84.5%)	52 (88.1%)	51 (91.1%)	17 (63%)
	31–35	16 (11.3%)	6 (10.2%)	2 (3.6%)	8 (29.6%)
	36–40	2 (1.4%)	1 (1.7%)	1 (1.8%)	0 (0%)
Level of training	Year 1	60 (42.3%)	14 (23.7%)	19 (33.9%)	27 (100%)
	Year 2	31 (21.8%)	12 (20.3%)	19 (33.9%)	0 (0%)
	Year 3–5	51 (35.9%)	33 (56.0%)	18 (32.2%)	0 (0%)
Characteristics of the training program			4–5 year duration	3-year duration	1-year duration
			60% of curriculum in the OT	90% of curriculum in the OT	95% of curriculum in the OT

### Design

**Fig. 1:**
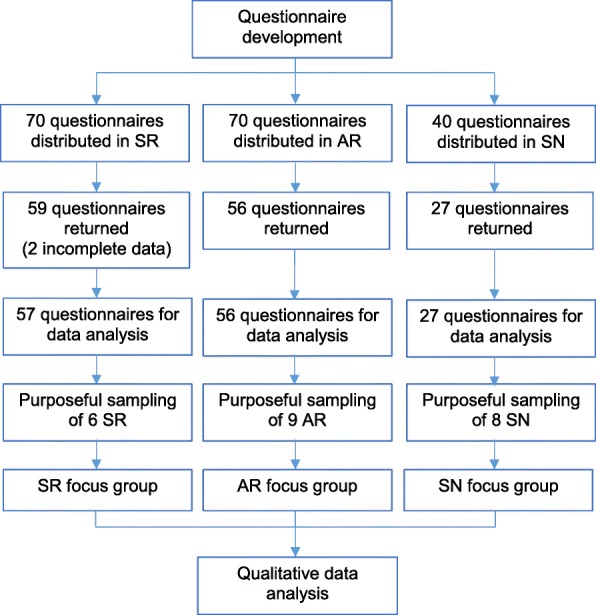
Study flow. SR = surgical residents; AR = anesthesia residents; SN = student nurse anesthetists

A mixed-methods methodology with an explanatory sequential design was used [25]. The explanatory sequential design was divided in two phases: quantitative and qualitative. For the first phase, we designed an instrument fit to evaluate the OT learning climate from the perspective of trainees from different professions. Next, all participants filled out the questionnaire about their perception of the learning climate in the OT. During the second phase mono-professional focus groups were utilized to explore the trainees’ perspectives of the survey results especially from the standpoints of different professions (Fig. 1).

### Quantitative phase

#### Instrument

The learning climate is an intellectual entity which is difficult to be quantitatively measured [10]. Because there was no previously-available instrument suitable for the operating theater setting and understandable to three different, professional groups (surgery, anesthesiology and nursing), a new questionnaire was created, based on a combination of three instruments aimed at measuring the educational environment: 1) the Dutch Residency Educational Climate Test (D-RECT) [22], 2) the Anesthetic Theatre Educational Environment Measure (ATEEM) [24], and 3) the Surgical Theatre Educational Environment Measure (STEEM) [23]. All three instruments have been previously validated and the permissions to reproduce all instruments were obtained. Items from each instrument were selected by comparing the three instruments to cover the main constructs of the three instruments and create a well-balanced instrument (acceptable in length, without redundancy and sufficient items per construct) that would be usable by trainees from all three professions.

This process resulted in the creation of a new questionnaire called the Operating Theater Educational Climate Test (OTECT), consisting of 46 items. Each item was assessed on a five-point Likert scale, where a score of 1 denoted ‘strongly disagree’ and a score of 5 denoted ‘strongly agree’.

The questionnaire was translated into Thai by two separate translators, followed by two separate retranslations back into English. The content validity of the Thai version was tested by piloting the questionnaire with surgical staff (*N* = 3), anesthesia staff (*N* = 3), nursing staff (*N* = 3) as well as trainees from all three groups (*N* = 15, 5 for each profession). Furthermore, a section was added to the questionnaire to collect demographic data from the participants (age, gender, professional department and level of study). Finally, trainees were asked to rate the overall quality of the learning climate in the operating theater, on a scale of one to five, as well as an open-ended question asking them to describe the factors they felt influenced the learning climate most. See Additional file 1: Appendix 1 for the questionnaire.

#### Data collection

The paper-based questionnaire was separately distributed to each group of participants during their morning conferences, topics of which ranged from a journal peer review and morbidity and mortality conferences once for each group. The study goals and the purposes of the study were presented to the participants via verbal communication and a brief, written outline at the beginning of the questionnaire. A research assistant distributed and collected the questionnaire without the primary investigator being present in the room.

#### Statistical analysis

##### Construct validity OTECT

To determine the construct validity of the newly designed questionnaire, an exploratory factor analysis was performed with Varimax rotation. The final amount of data for factor analysis was 140 (excluding missing data), providing a ratio of over three cases per variable. The Kaiser-Meyer-Olkin measure of sampling adequacy was 0.896, which was above the commonly recommended value of 0.6, and Bartlett’s test of sphericity was significant (*p* < 0.001). The number of factors was forced to 9 from the original version of the questionnaire that contained 9 factors [26]. Two factors were then eliminated due to them containing items with double loading. Furthermore the Cronbach’s alpha coefficients were calculated to determine the internal consistency of the scales.

##### Demographic data

Descriptive statistics were performed on the demographic data.

##### OTECT items

Items with negative phrasing were recoded before analyzing the data. The Likert-scale items were analyzed and presented as a mean and a standard deviation and analyzed per professional group. Thereafter analyses were performed on the factor level for each group. To look for differences between professional groups analysis of variance (ANOVA) was performed. The Bonferoni correction was then performed for post-hoc analysis to minimize the type 1 error [27]. The significant difference was determined at the *p* < 0.05 levels.

### Qualitative phase

#### Data collection

Following the Consolidated criteria for reporting qualitative research [28], three mono-professional focus groups were organized during December 2014 and January 2015 to further explore and understand the findings from the quantitative phase. Participants were purposively sampled so that each group consisted of trainees situated in each department belonging to a profession were represented as well as trainees from different training levels. [29]. Groups ranged in size from six to nine participants each (six surgical residents, nine anesthesia residents, and eight student registered-nurse anesthetists), and the discussions lasted from 65 to 85 min. Because a member of the teaching staff might inadvertently influence the trainees’ expression of ideas during the discussions, possible bias was reduced by having each focus group moderated by a non-teaching staff member, a female nurse, who had been previously trained in focus group facilitation.

Written informed consent was also obtained from all participants before conducting the focus groups. The purpose of the qualitative phase was conveyed to all participants during the recruitment process and at the beginning of the discussion.

The moderator used a semi-structured discussion guide (Additional file 1: Appendix 2) to stimulate participants’ responses. The moderators followed the guide to initiate the discussion, but retained the flexibility to modify the question order when the flow of the conversation made sense to do so.

#### Data analysis

The verbatim transcripts were independently coded and analyzed by the principal investigator and an educational scientist who has experience with qualitative analysis in educational research. Analysis firstly followed principles of open coding in order to get a general appreciation of the data and its meaning. The meaningful text relevant to the research question was highlighted and coded. The codes were subsequently conceptualized and used to create themes. Axial coding was then performed to identify the relationships among the themes. Any discrepancies in the themes and codes were discussed until both researchers obtained consensus. Member checking was performed by discussing the results with a random selection of the participants.

#### Ethical approval

The local IRB (Siriraj Institutional Review Board, Bangkok, Thailand) approved the study protocol before data collection proceeded (Si561/2014).

## Results

### Quantitative: questionnaire

Of the 180 questionnaires distributed, 142 were returned of which two incomplete, which were removed before analysis. This resulted in a response of 77.8%. Respondent characteristics are presented in Table 1.

#### Construct validity OTECT

Factor analysis was performed to identify and compute composite scores for the factors. Thirteen items were eliminated from the analysis because they had double-factor loading or were theoretically irrelevant to other items within the scale [26] as shown in Additional file 1: Appendix 3. The exploratory factor analysis resulted in a seven-factor model explaining 66.2% of the variance (see Table 2). The seven distinct factors were titled *supervisor roles*, *independent practice, assessment and evaluation, interprofessional collaboration, feedback forms, presence of negative attitudes towards trainees, and personal well being of student*. These factors were comparable to the original questionnaires: ‘supervisor roles’ and ‘assessment and evaluation’, were derived from the D-RECT, ‘independent practice’ originated from the ATEEM. A minor change of scales occurred in the original scale of ‘perception of atmosphere’ (originating from STEEM and ATEEM), which was split into two scales of ‘interprofessional collaboration’ and ‘presence of negative attitudes toward student’.

**Table 2:** Factor loadings and communalities based on a principal components analysis with Varimax rotation for 33 items from the questionnaire and the Cronbach alpha of each scale

Rotated Component Matrix^a^							
	Component						
	1	2	3	4	5	6	7
1. Supervisor Roles (α = 0.89)							
32. My supervisors are all in their own way positive role models	.735						
34. When I need to consult a supervisor, they are readily available	.725						
28. My supervisors are happy to discuss patient care	.663						
29. There is (are) NO supervisor(s) who have a negative impact on the educational climate	.663						
31. My supervisors treat me with respect	.654						
33. When I need a supervisor, I can always contact one	.635						
27. My supervisors take time to explain things when asked for advice	.594						
2. Independent practice (α = 0.89)							
45. I have the opportunity to acquire the appropriate practical procedures for my level of training		.819					
44. I have an appropriate level of clinical responsibility		.810					
42. I feel responsible and accountable for the care given to my patients		.730					
41. There is a clinical training programme here that allows me to get first-hand experience in a range of procedures		.692					
40. I am clear about the learning objectives of the theatre teaching session		.576					
43. I am aware of my professional role in the theatre		.547					
3. Assessment & Evaluation (α = 0.87)							
13. My supervisors take the initiative to evaluate my performance			.729				
14. My supervisors take the initiative to evaluate difficult situations I have been involved in			.685				
12. My supervisors take the initiative to explain their actions			.665				
16. My supervisors assess not only my medical expertise but also other skills such as teamwork, organization or professional behavior			.593				
11. I am asked on a regular basis to provide a rationale for my management decisions and actions			.576				
15. My supervisors evaluate whether my performance in patient care is commensurate with my level of training			.545				
4. Interprofessional collaboration (α = 0.88)							
2. The people I work with are friendly				.791			
1. I have good collaboration with theater staff				.787			
3. The atmosphere in theatre is pleasant				.687			
6. I feel part of a team in theatre				.671			
8. Other allied health professionals make a positive contribution to my training				.606			
7. Attendings, nursing staff, other allied health professionals and residents work together as a team				.569			
9. Other allied health professionals are willing to reflect with me on the delivery of patient care				.563			
10. Teamwork is an integral part of my training				.559			
5. Feedback forms (α = 0.94)							
19. Observation forms (i.e., Mini-CEX) are used periodically to monitor my progress					.856		
18. Observation forms (i.e., Mini-CEX) are used to structure feedback					.838		
6. Presence of negative attitudes towards student (α = 0.62)							
4. The staff from other discipline dislike it when I practice my skills as the procedure takes longer*						.737	
5. I feel discriminated against in theatre because of my sex*						.692	
7. Personal well-being of student (α = 0.64)							
39. I am so stressed in theatre that I do not learn as much as I could*							.748
38. I am often too tired to get the most out of theatre teaching*							.719

Extraction Method: Principal Component Analysis

Rotation Method: Varimax with Kaiser Normalization

^a^Rotation converged in 16 iterations.

Factor loading < 0.4 are suppressed.

Items with * have negative phrasing were recoded before the statistical analysis

Cronbach’s alpha analysis resulted in a normal alpha of all items of 0.95, with the standardized items alpha of 0.96, which means the reliability of the questionnaire was high. The internal consistency for each scale was in acceptable range and revealed no item that causes substantial decrease in alpha within the scale if the item was removed (see Table 2).

#### Results OTECT

Overall, all respondent groups rated the OT learning climate as relatively positive (mean = 3.63 (0.70)) and the overall quality similarly (see Additional file 1: Appendix 4 for item level results). All groups rated the supervisor role as highest (mean = 4.23 (0.67) see Table 3) whereas personal wellbeing of student was rated lowest (mean = 3.00 (0.78)).

**Table 3:** Mean score (±SD) on the factor level for each group and the one-way ANOVA comparing the difference between groups

Factors	Overall mean scores (±SD) *N* = 140	SR mean scores (±SD) *N* = 57	AR mean scores (±SD) *N* = 56	SN mean scores (±SD) *N* = 27	F	Sig
Supervisor role	4.23 (±0.67)	4.18 (±0.80)	4.19 (±0.56)	4.40 (±0.52)	1.14	0.32
Independent practice	3.80 (±0.54)	3.72 (±0.65)	3.80 (±0.43)	3.96 (±0.42)	1.99	0.14
Assessment and evaluation	3.75 (±0.50)	3.73 (±0.61)	3.71 (±0.39)	3.89 (±0.45)	1.31	0.27
Interprofessional collaboration	3.81 (±0.51)	3.81 (±0.64)	3.75 (±0.40)	3.92 (±0.41)	0.93	0.40
Feedback forms	3.21 (±0.76)	3.05 (±0.87)	3.20 (±0.56)	3.58 (±0.75)	4.73	0.01*
Presence of negative attitudes towards student	3.19 (±0.88)	3.14 (±0.99)	3.02 (±0.78)	3.67 (±0.62)	5.47	0.005*
Personal well-being of student	3.00 (±0.78)	3.18 (±0.91)	3.01 (±0.58)	2.61 (±0.75)	5.02	0.008*
Overall quality	3.63 (±0.70)	3.59 (±0.82)	3.59 (±0.63)	3.81 (±0.56)	1.15	0.32

* the mean difference is significant at the 0.05 level; *SR* surgical residents, *AR* anesthesia residents, *SN* student nurse anesthetists

Respondent groups differed significantly in their evaluation of the factors ‘feedback forms’, ‘presence of negative attitude towards trainees’ and ‘personal well-being of trainees’ demonstrated by one-way ANOVA with Bonferoni post-hoc analysis at the *p* < 0.05 levels (Table 3). The factor ‘feedback forms’ was rated lowest by the surgical residents (SR). Respondents from the AR group rated the ‘presence of negative attitudes towards student’ lowest. Overall, the wellbeing of student registered-nurse anesthetists (SN) in the OT was the lowest (mean score = 2.61 (0.75)).

### Qualitative: focus groups

Questionnaire results were discussed during three mono-professional focus groups. Three main themes were constructed from the discussion by participants from each focus group: 1) the nature of work in the OT, 2) the role of the supervisors, and 3) the interprofessional dimension of working in an OT. There were minor differences in how participants described each theme, but the overall picture was the same for all three groups.

## Theme 1: The nature of work in an OT

Characteristics of the OT work environment were the main factor discussed by participants in all three groups. Mainly influencing their learning was 1) the high-stakes nature of the OT and how supervisors deal with it, 2) working with time constraints, and 3) physical discomfort caused by being in the OT.

### The high stake nature of the OT and how supervisors deal with it

All participants indicated that the nature of the learning environment, namely high stakes, negatively influenced the learning experience in the OT. Patient safety always came first, backgrounding their learning process. One anesthesia resident said:

“*It is a place where we put a patient between life and death. Yes, we want to learn, but the patient safety always comes first.*” (Anesthesia resident, third year.)

All participants mentioned that how their supervisors dealt with critical situations heavily influenced their learning experiences and the extent to which they felt that the supervisor was a good role-model.

“*Once, one of my supervisors used bad words and yelled in my face during a critical period. I know that he was stressed out because he was responsible for the patient safety. But to be honest, I was shocked; I had never expected anyone to act like this in such a tense situation.*” (Student nurse anesthetist, first year.)

### Working with time constraints

How the OT is organized can hinder learning by putting the importance of service ahead of education. The service burden led to a condensed operative list and an increased workload for trainees. It also resulted in a time-constraint issue in the OT as a place to learn. All participants recognized this problem and reported it as affecting their learning. The pressure to utilize resources efficiently combined with tight operating schedules resulted in a sense of continuous rush and hurriedness. Feeling rushed forced the learners to keep up with the OT team instead of taking the time to elaborate and reflect on the knowledge gained and to practice their skills.

“….*like when I sent the patient off from the OT to the recovery room, this should be a part of learning to look after the patient in the recovery room as an immediate postoperative care, right? But the next patient on the list had been wheeled in the operating room right after the previous patient was out. And I was the last one running back to the OT with everybody waiting and looking at me as if I was the one who held the team back.*” (Anesthesia resident, first year.)

Participants from AR and SN groups reported that hurriedness sometimes came from co-workers who did not wait for them to practice their skills. This was not an issue in the SR group, who recognized their role and tried to strike a balance between education and service. One participant from the SN group mentioned:

“*Some of the co-workers can be very pushy. They don’t want to wait for me to get my job done. Like one time after intubation, I didn’t even put a tape on the tube, but they were ready to turn the patient prone.”* (Student nurse anesthetist, first year.)

### Physical discomfort caused by being in the OT

The physical discomfort caused by room temperature and the large amount of sensory information was discussed by all groups. However, participants identified different elements of the physical environment causing discomfort. One participant from the SN group reported that the noisy environment hampered meaningful contact with her supervisor:

“*Sometimes, the OT can be very noisy, like the sound of the drilling and hammering in orthopedic theater. I understand that it is unavoidable. But it disturbed me when I tried to elaborate on my discussion with the supervisor.*” (Student nurse anesthetist, first year.)

Participants from the AR group did not mention the noise, but had an issue with the temperature: too cold an operating room made them feel uncomfortable. In contrast, one participant from the SR group reported that quietness was a problem that could raise the stress level of the student; as she said:

“*I would like it if my supervisor plays music during the operation, any kind of music would do. It makes the overall environment positive, including learning environment. Imagine when the room is so quiet, and a tense situation occurs, like massive bleeding, the tension in the air will be magnified when it’s too quiet.*” (Surgical resident, fourth year.)

## Theme 2: The Role of the Supervisors

Participants in all three groups intensively and elaborately discussed the role of the supervisor. Particularly 1) diverse supervisor demands, and 2) the unsafe learning climate that was sometimes created by supervisors, came forward during discussion.

### Diverse supervisor demands

As a result of the high-stakes environment, supervisors could be very demanding regarding upholding their own standards for patient safety. Each supervisor had his or her own techniques, which might not correspond with their colleagues’ techniques. This sometimes resulted in an atmosphere of disrespect among supervisors, which was felt to be unpleasant by the postgraduate trainees and specifically described by AR and SN participants. Participants were set on socializing within the environment and as such tried to adapt to the best of their abilities to the demands of the various supervisors.

“*I thought the anesthetizing style of supervisor A was great, so I used this style when I was working with supervisor B. I knew that there was nothing wrong with this technique. But it just was not supervisor B’s style, and she said… I am not that person; working with me, you have to go my way.*” (Anesthesia resident, third year.)

However, at the same time exposure to different approaches was also described as a positive aspect of a learning environment by all three groups: it provided alternative approaches to looking after patients.

### Unsafe learning climate

Participants from all groups also discussed negative behavior by supervisors and how it could influence the entire learning climate. Being scolded lowered student’s self-esteem and impacted on their long-term relationship with the supervisors. This could also lead to a feeling of fear and loss of autonomy among the trainees. The effect on student’s feelings was strong and hurtful. One surgical resident reported:

“*After being scolded in the OT, I was so stressed that I didn’t want to learn anything. I just wanted these two hours to end very quickly. I didn’t even know what the operation was all about. I just wanted to concentrate on my surgical assistant job, like cut the suture equally, hold the retractor still, to avoid being scolded again.*” (Surgical resident, fourth year.)

An unsafe learning climate also undermined the dignity of the trainees and affected their relationship with patients. One participant from the AR group said:

“*My worst experience was when I was scolded in front of the patient. I knew I was doing something critically wrong at that moment, but it ruined my patient’s respect and trust and made me lose my self-esteem.*” (Anesthesia resident, first year.)

## Theme 3: Interprofessional dimension to the OT

Working and learning with members of other professions is an important characteristic of the OT. All professionals being supportive of each other’s learning is an important factor in fostering learning by creating a positive learning environment.

“*I like when the ‘big’ surgeon sees me as a person. I always feel like I’m a small, humble nurse. Some surgeons treat me with respect and discuss the positioning of the patients or potential complications with me. Those make me feel so good*.” (Student nurse anesthetist, first year.)

Participants from all groups regarded good interprofessional team communication and collaboration as a factor positively affecting the learning environment. Good team communication provided interprofessional collaborative learning.

The other factor raised by the SR group was learning to work interprofessionally. The participants learned the importance of teamwork in promoting a positive learning climate. One surgical resident said:

“*It’s important to work as a team. I’m the surgeon, but I cannot operate without the team. It seems to be the same in terms of learning. Learning together with other professionals is more effective in workplace settings, like discussing the plan for this patient together*.” (Surgical resident, fourth year.)

## Discussion

The influence of the learning climate on outcomes of post-graduate medical education has been widely recognized [5, 9]. By evaluating the learning climate, we can get insights in how to improve student learning but also how to (re)design the learning environment and support faculty in their teaching role [21]. Traditionally the experienced learning climate has been studied ‘mono-professionally’: from the perspective of trainees within one profession. However, the OT is a learning climate for trainees of diverse professions. Therefor this study set out to understand how three groups of trainees from different professions experienced the learning climate within one working environment, the OT.

To this purpose we developed the OTECT questionnaire, which was found to be a valid instrument (content and construct validity) and has therefor given us a way to evaluate the OT learning climate from the perspective of all postgraduate trainees present in that environment. Both the results of the OTECT and focus groups indicated similar perceptions of the learning climate for all groups of learners, however some differences and nuances in their perceptions and experiences were also noted. Focus group discussions provided additional depth to the OTECT findings and pointed to the importance of the high stakes nature of the OT, the supervisor, and the interdisciplinary nature of collaborating in the OT to the experienced learning climate.

### Positive or negative learning climate?

The OTECT results demonstrated that the overall learning climate in the OT was experienced as positive for all groups from different professions, which is in line with the survey results of Boor et al. [22] and Silkens et al. [11] studies. The relatively low scores on the personal wellbeing subscale from this study were comparable to the results found by Holt and Roff [24] and may be due to the characteristic of postgraduate training programs in health professions with substantial levels of workload that can negatively effect trainees’ wellbeing [30]. When looking at the separate subscales, the supervisor factors, interprofessional collaboration and independent practice were rated highest by all trainee groups, potentially implying that these factors can support learning in the OT for all groups. When comparing the OTECT results between groups, the feedback forms and presence of negative attitudes towards trainees were significantly better in the SN group. This may be due to the nature of the SN program where trainees have less responsibility in clinical work and can therefore focus more on learning than their surgery and anesthesiology colleagues. The focus group results pointed out the importance of interprofessional learning among the participants, which is in line with the questionnaire results. However, focus group results highlighted how supervisors might actually hinder trainee learning. This renders the question to what the OTECT stimulates more positive than negative answers about the supervisor role in the educational climate.

This question becomes even more pertinent when comparing the OTECT results to the focus groups: While the OTECT results pointed to a mainly positive learning climate, the focus group discussions were dominated by how the participants experienced a negative learning climate. This demonstrates the potential of a Mixed Methods Methodology to unveil different parts of the same picture [25] and the opportunity provided by the focus groups to dive deeper into and better understand participants experience [29].

### Supervision & high stakes environment

The results of the OTECT questionnaires and the focus groups both pointed to the importance of the supervisor in the OT for all three groups: Supervisors could make or break the learning climate. The key role of the supervisor in the clinical learning environment is supported by a large body of literature from different health professional contexts and levels of training [31–34]. Olmos-Vega [35, 36] and Marshall [37] revealed that balancing between clinical supervision and resident autonomy according to level of training is an important factor to improve residents’ preferences, which could affect the experienced learning climate. Iwaszkiewicz et al. [38] explored the characteristics of supervisors who were considered good supervisors in the OT as perceived by surgical residents. Good supervisors created a favorable learning climate by being polite and respectful to their colleagues and trainees, staying calm during crisis situations, providing constructive feedback and demonstrating enthusiasm to teach. Our results add to these findings that inappropriate stress-coping mechanisms of supervisors during crisis as well as the impolite and unsafe learning climate sometimes created strongly impact the learning experiences of trainees in that environment.

The high-stakes OT environment affected supervisors and trainees from all three professional groups. Because patient care and safety are the primary goals of the OT, supervisors need to constantly weigh how to ensure patient safety while creating learning opportunities [37]. Our focus groups results revealed that postgraduate trainees were very aware of the importance of patient safety and did put it ahead of their learning. However, sometimes supervisors responded to high-stakes situations by creating an unsafe learning climate by criticizing, belittling and scolding. Participants from all groups recognized that an unsafe learning climate could be generated by the verbal and nonverbal behavior of their supervisors. Both Lyon [39] and Flott and Linden [40] described how the high-stakes nature of the OT environment directly influenced trainees’ ability to learn there. Lyon more specifically indicated how learning in the OT requires a balance between the emotional impact of the high-stakes environment, the purpose of educational tasks in the busy OT, and the social and interprofessional relations [39]. Ali et al. [41] and Bragard et al. [42] studied the stress level of emergency physicians and found high levels of stress caused by the nature of high stake environment as well as the need to supervise trainees. Dependent on the supervisor’s coping style, their expressions of emotion and stress could directly influence the postgraduate trainees. Our study demonstrated that when the supervisors had differing preferences and demanded that their personal preferences were followed, it affected the trainees by creating feelings of ambiguity and hampering their experienced learning climate. The participants in our study coped with the unsafe learning environment by making their learning secondary and being as unobtrusive as possible – they avoided critical thinking, discussing and practicing – in order to avoid worsening the atmosphere. Hoel et al. [43] point to the fact that it is important to consider how negative feelings caused by supervisors influence the socialization process and potentially stimulates reproduction of this behavior in trainees, producing a chain of unfriendly and negative learning atmosphere in the clinical setting.

### Time pressure and physical discomfort

Although all three student groups in our study experience the OT as an environment characterized by rushing and hurriedness, how this influenced their learning seemed to differ. The surgical residents’ views on the time constraint issue was different from other groups but consistent with previous work by Vikis et al. [13]. Surgical residents revealed that they found a balance between learning and time constraints. While the anesthesia residents and nurse trainees in our study revealed the pressure from time constraint affected their clinical learning. Also physical discomfort from the OT environment, such as cold and noise, was found to be a factor inhibiting trainees’ ability to learn. Pilcher et al. [44] reported the effect of the environment’s temperature on the cognitive function of trainees: being in too cold an environment impairs learning capability and student performance, and the duration of the exposure to the cold environment also has consequences for performance. By comparison, participants from this study found that both noise and quietness influenced their learning climate. The physical environment appeared to be an important and simple element that could be easily modified to make it more suitable for student learning [40].

### Limitations of the study and suggestions for future research and practice

This is a single-center study, which limits the transferability of the results. However, by providing a specific description of the context and participants of this study and by grounding the work in theories of workplace-based learning and the learning climate we hope to have augmented the transferability. We need to note that although the OT environment is reasonably homogeneous even in various countries, this study was conducted in Thailand, which is a relative high power distance according to Hofstede’s cultural dimensions [45]. Future research needs to focus on how professional culture and national culture interact when it comes to learning climate.

The sample population is also limited in number. The construct validation process ideally requires more respondents to fully determine the validity of the questionnaire. However, from a perspective of pragmatism [26] the newly developed OTECT was useful for the purpose of understanding how postgraduate trainees from different professions experience the same learning climate.

Future research could look into the effects of an interprofessional learning environment in other clinical settings to help enhance interprofessional collaboration and improve the educational environment in order to maximize the effectiveness of workplace-based leaning for postgraduate trainees from all professions present within the same setting. The newly designed OTECT might assist program leaders in diagnosing the learning climate within their OR’s and improve the learning experiences of their trainees within the OT.

## Conclusions

The operating theater is the main learning environment for various trainees. Currently, there is no published study comparing the perceptions of the learning climate in the OT for different professional postgraduate trainees such as student nurses, surgical residents and anesthesia residents. This study of our study revealed that they all had comparable experience in this learning environment. The learning climate in the OT is mainly hindered by the high stakes nature of the environment and the effect it has on the supervisors and the postgraduate trainees. The results can be applied to improve the overall learning environment in the OT for all groups of learners. Future research should try to optimize the learning environment without compromising the patient safety.

## Additional file

Additional file 1: **Appendix 1.** OTECT Questionnaire. **Appendix 2.** Focus group discussion guide. **Appendix 3.** Eliminated items from principle component analysis. **Appendix 4.** Mean scores and standard deviation of the response from the questionnaires. (DOCX 138 kb)

## Data Availability

All relevant data are available in the manuscript and appendices. The complete datasets generated and/or analysed during this current study are available from the corresponding author on reasonable request.

## References

[1] Genn JM (2001). AMEE medical education guide no. 23 (part 1): curriculum, environment, climate, quality and change in medical education-a unifying perspective. Med Teach..

[2] Genn JM (2001). AMEE medical education guide no. 23 (part 2): curriculum, environment, climate, quality and change in medical education-a unifying perspective. Med Teach..

[3] Dornan T, Boshuizen H, King N, Scherpbier A (2007). Experience-based learning: a model linking the processes and outcomes of medical students' workplace learning. Med Educ.

[4] Stalmeijer RE, Dolmans DH, Snellen-Balendong HA, van Santen-Hoeufft M, Wolfhagen IH, Scherpbier AJ (2013). Clinical teaching based on principles of cognitive apprenticeship: views of experienced clinical teachers. Acad Med.

[5] Chan DS (2002). Associations between student learning outcomes from their clinical placement and their perceptions of the social climate of the clinical learning environment. Int J Nurs Stud.

[6] Weiss KB, Bagian JP, Nasca TJ (2013). The clinical learning environment: the foundation of graduate medical education. JAMA..

[7] Smirnova A, Ravelli AC, Stalmeijer RE, Arah OA, Heineman MJ, van der Vleuten CP, van der Post JA, Lombarts KM (2017). The association between learning climate and adverse obstetrical outcomes in 16 nontertiary obstetrics–gynecology departments in the Netherlands. Acad Med.

[8] Wagner R, Weiss KB, Passiment ML, Nasca TJ (2016). Pursuing excellence in clinical learning environments. J Grad Med Educ.

[9] Silkens ME, Lombarts KM, Scherpbier AJ, Heineman MJ, Arah OA (2017). Towards healthy learning climates in postgraduate medical education: exploring the role of hospital-wide education committees. BMC Med Educ.

[10] Soemantri D, Herrera C, Riquelme A (2010). Measuring the educational environment in health professions studies: a systematic review. Med Teach..

[11] Silkens ME, Chahine S, Lombarts KM, Arah OA (2018). From good to excellent: improving clinical departments’ learning climate in residency training. Med Teach..

[12] Makary MA, Sexton JB, Freischlag JA, Holzmueller CG, Millman EA, Rowen L, Pronovost PJ (2006). Operating room teamwork among physicians and nurses: teamwork in the eye of the beholder. J Am Coll Surg.

[13] Vikis EA, Mihalynuk TV, Pratt DD, Sidhu RS (2008). 2008. Teaching and learning in the operating room is a two-way street: resident perceptions. Am J Surg.

[14] Cardoen B, Demeulemeester E, Beliën J (2010). Operating room planning and scheduling: a literature review. Eur J Oper Res.

[15] Bleakley A (2006). A common body of care: the ethics and politics of teamwork in the operating theater are inseparable. J Med Philos.

[16] Bleakley A, Boyden J, Hobbs A, Walsh L, Allard J (2006). Improving teamwork climate in operating theatres: the shift from multiprofessionalism to interprofessionalism. J Interprof Care.

[17] Lingard L, Reznick R, Espin S, Regehr G, DeVito I (2002). Team communications in the operating room: talk patterns, sites of tension, and implications for novices. Acad Med.

[18] Rees CE, Crampton P, Kent F, Brown T, Hood K, Leech M, Newton J, Storr M, Williams B (2018). Understanding students’ and clinicians’ experiences of informal interprofessional workplace learning: an Australian qualitative study. Br Med J Open.

[19] Varpio L, Bidlake E, Casimiro L, Hall P, Kuziemsky C, Brajtman S, Humphrey-Murto S (2014). Resident experiences of informal education: how often, from whom, about what and how. Med Educ.

[20] Weiss KB, Co JPT, Bagian JP (2018). Challenges and opportunities in the 6 focus areas: CLER National Report of findings 2018. J Grad Med Educ..

[21] Lombarts KM, Heineman MJ, Scherpbier AJ, Arah OA (2014). Effect of the learning climate of residency programs on Faculty's teaching performance as evaluated by residents. PLoS One.

[22] Boor K, Van Der Vleuten C, Teunissen P, Scherpbier A, Scheele F (2011). Development and analysis of D-RECT, an instrument measuring residents' learning climate. Med Teach..

[23] Cassar K (2004). Development of an instrument to measure the surgical operating theatre learning environment as perceived by basic surgical trainees. Med Teach..

[24] Holt MC, Roff S (2004). Development and validation of the Anaesthetic theatre educational environment measure (ATEEM). Med Teach.

[25] Creswell JW, Creswell JW (2012). Mixed methods designs. Educational research. Planning, conducting and evaluating quantitative and qualitative research.

[26] Field A, Field A (2013). Exploratory factor analysis. Discovering statistics using SPSS.

[27] Hilton A, Armstrong R. Stat note 6: Post hoc ANOVA tests. Microbiologist. 2006;34–36.

[28] Tong A, Sainsbury P, Craig J (2007). Consolidated criteria for reporting qualitative research (COREQ): a 32-item checklist for interviews and focus groups. Int J Qual Health Care.

[29] Stalmeijer RE, McNaughton N, Van Mook WN (2014). Using focus groups in medical education research: AMEE guide no. 91. Med Teach..

[30] Jennings ML, Slavin SJ (2015). Resident wellness matters: optimizing resident education and wellness through the learning environment. Acad Med.

[31] Spencer J (2003). Learning and teaching in the clinical environment. Br Med J.

[32] Ramani S, Leinster S (2008). AMEE guide no. 34: teaching in the clinical environment. Med Teach..

[33] Schwind CJ, Boehler ML, Rogers DA, Williams RG, Dunnington G, Folse R, Markwell SJ (2004). Variables influencing medical student learning in the operating room. Am J Surg.

[34] Meyer R, Van Schalkwyk SC, Prakaschandra R (2016). The operating room as a clinical learning environment: an exploratory study. Nurs Educ Pract.

[35] Olmos-Vega FM, Dolmans DH, Donkers J, Stalmeijer RE (2015). Understanding how residents’ preferences for supervisory methods change throughout residency training: a mixed-methods study. BMC Med Educ.

[36] Olmos-Vega FM, Dolmans DH, Vargas-Castro N, Stalmeijer RE (2017). Dealing with the tension: how residents seek autonomy and participation in the workplace. Med Educ.

[37] Marshall SD (2015). Sink or swim? The difficulty of finding the correct level of independence and support for trainees. Br J Anaesth.

[38] Iwaszkiewicz M, DaRosa DA, Risucci DA (2008). Efforts to enhance operating room teaching. J Surg Educ.

[39] Lyon P (2003). Making the most of learning in the operating theatre: student strategies and curricular initiatives. Med Educ.

[40] Flott EA, Linden L (2016). The clinical learning environment in nursing education: a concept analysis. J Advanc Nurs.

[41] Ali S, Thomson D, Graham TA, Rickard SE, Stang AS (2017). High stakes and high emotions: providing safe care in Canadian emergency departments. Open Access Emerg Med.

[42] Bragard I, Dupuis G, Fleet R (2015). Quality of work life, burnout, and stress in emergency department physicians: a qualitative review. Eur J Emerg Med.

[43] Hoel H, Giga SI, Davidson MJ (2007). Expectations and realities of student nurses' experiences of negative behaviour and bullying in clinical placement and the influences of socialization processes. Health Serv Manag Res.

[44] Pilcher JJ, Nadler E, Busch C (2002). Effects of hot and cold temperature exposure on performance: a meta-analytic review. Ergonomics..

[45] Hofstede G (1984). Cultural dimensions in management and planning. Asia Pac J Manag.

