# Supplementary material for: How postgraduate trainees from different health professions experience the learning climate within an operating theater: a mixed-methods study
Source: BMC Med Educ. 2019 Jun 21;19:221. doi: 10.1186/s12909-019-1648-1 (PMC6588845; doi:10.1186/s12909-019-1648-1)
Supplement: Supplementary file 1 — Appendix 1. OTECT Questionnaire. Appendix 2. Focus group discussion guide. Appendix 3. Eliminated items from principle component analysis. Appendix 4. Mean scores and standard deviation of the response from the questionnaires. (DOCX 138 kb) [file 12909_2019_1648_MOESM1_ESM.docx]

#### Appendix 1 OTECT Questionnaire

*Measuring the educational environment in the operating theater*

| *Items* | *Original version* | *Original Tool* |
| --- | --- | --- |
| *Perceptions of atmosphere* |  |  |
| 1. I have good collaboration with theatre staff |  | ATEEM |
| 1. The people I work with are friendly |  | ATEEM |
| 1. The atmosphere in theatre is pleasant |  | STEEM |
| 1. The staff from other discipline dislike it when I practice my skills as the procedure takes longer* | The nursing staff dislike it when I operate as the operation takes longer | STEEM |
| 1. I feel discriminated against in theatre because of my sex* |  | STEEM |
| 1. I feel part of a team in theatre |  | STEEM |
| *Teamwork* |  |  |
| 1. Attendings, nursing staff, other allied health professionals and residents work together as a team |  | D-RECT |
| 1. Other allied health professionals make a positive contribution to my training | Nursing staff and other allied health professionals make a positive contribution to my training | D-RECT |
| 1. Other allied health professionals are willing to reflect with me on the delivery of patient care | Nursing staff and other allied health professionals are willing to reflect with me on the delivery of patient care | D-RECT |
| 1. Teamwork is an integral part of my training |  | D-RECT |
| *Coaching and assessment* |  |  |
| 1. I am asked on a regular basis to provide a rationale for my management decisions and actions |  | D-RECT |
| 1. My supervisors take the initiative to explain their actions | My attendings take the initiative to explain their actions | D-RECT |
| 1. My supervisors take the initiative to evaluate my performance | My attendings take the initiative to evaluate my performance | D-RECT |
| 1. My supervisors take the initiative to evaluate difficult situations I have been involved in | My attendings take the initiative to evaluate difficult situations I have been involved in | D-RECT |
| 1. My supervisors take the initiative to evaluate difficult situations I have been involved in | My attendings evaluate whether my performance in patient care is commensurate with my level of training | D-RECT |
| 1. My supervisors assess not only my medical expertise but also other skills such as teamwork, organization or professional behavior | My attendings assess not only my medical expertise but also other skills such as teamwork, organization or professional behavior | D-RECT |
| *Feedback* |  |  |
| 1. My supervisors give regular feedback on my strengths and weaknesses | My attendings give regular feedback on my strengths and weaknesses | D-RECT |
| 1. Observation forms (i.e., Mini-CEX) are used to structure feedback |  | D-RECT |
| 1. Observation forms (i.e., Mini-CEX) are used periodically to monitor my progress |  | D-RECT |
| *Professional relations between supervisors* |  |  |
| 1. Continuity of care is not affected by differences of opinion between supervisors | Continuity of care is not affected by differences of opinion between attendings | D-RECT |
| 1. Differences of opinion between supervisors about patient management are discussed in such a manner that is instructive to others present | Differences of opinion between attendings about patient management are discussed in such a manner that is instructive to others present | D-RECT |
| 1. Differences of opinion are not such that they have a negative impact on the work climate |  | D-RECT |
| Work is adapted to residents’ competence |  |  |
| 1. The work I am doing is commensurate with my level of experience |  | D-RECT |
| 1. The work I am doing suits my learning objectives at this stage of my training |  | D-RECT |
| 1. It is possible to do follow up with patients |  | D-RECT |
| 1. There is enough time in the schedule for me to learn new skills |  | D-RECT |
| *Supervisors’ role* |  |  |
| 1. My supervisors take time to explain things when asked for advice | My attendings take time to explain things when asked for advice | D-RECT |
| 1. My supervisors are happy to discuss patient care | My attendings are happy to discuss patient care | D-RECT |
| 1. There is (are) NO supervisor(s) who have a negative impact on the educational climate | There is (are) NO attending physician(s) who have a negative impact on the educational climate | D-RECT |
| 1. My supervisors treat me as an individual | My attendings treat me as an individual | D-RECT |
| 1. My supervisors treat me with respect | My attendings treat me with respect | D-RECT |
| 1. My supervisors are all in their own way positive role models | My attendings are all in their own way positive role models | D-RECT |
| 1. When I need a supervisor, I can always contact one | When I need a attending, I can always contact one | D-RECT |
| 1. When I need to consult a supervisor, they are readily available | When I need to consult an attending, they are readily available | D-RECT |
| 1. The amount of supervision I receive is appropriate for my level of experience |  | D-RECT |
| 1. I discuss the management plan of cases with the theatre teacher |  | ATEEM |
| *Workload and support* |  |  |
| 1. Whenever I should participate in formal educational  programmes I get relief from theatre duties |  | ATEEM |
| 1. I am often too tired to get the most out of theatre teaching* |  | STEEM |
| 1. I am so stressed in theatre that I do not learn as much as I could* |  | STEEM |
| *Autonomy* |  |  |
| 1. I am clear about the learning objectives of the theatre teaching session |  | ATEEM |
| 1. There is a clinical training programme here that allows me to  get first-hand experience in a range of procedures |  | ATEEM |
| 1. I feel responsible and accountable for the care given to my patients |  | ATEEM |
| 1. I am aware of my professional role in the theatre | I am aware of my anaesthetic role in theatre | ATEEM |
| 1. I have an appropriate level of clinical responsibility |  | ATEEM |
| 1. I have the opportunity to acquire the appropriate practical  procedures for my level of training |  | ATEEM |
| *Overall quality* |  |  |
| 1. The overall educational environment in the OR is appropriate |  |  |

* Items in negative phrases

47. Which factors contribute most to the overall quality of learning climate in the OR? ____________________________________________________________________________________________________________________________________________________________________________________________________________________________________________________________

#### Appendix 2: Focus group discussion guide

Focus Group Discussion Guide

1. Consent process & introduction

We are very pleased you have agreed to join us today. We are here to talk about the perceptions of the postgraduate trainees of the learning climate in the operating room.

The discussion we are going to have is called a focus group. For those of you who have never participated in one of these sessions I would like to explain a little bit about this type of research.

Focus groups are used to gather information informally from a small group of individuals who have a common interest in a particular subject, in this case, you are all postgraduate trainees who mainly learn and practice in the OR.

This discussion will provide information about learning climate on the OR for further improvement in the future.

In focus groups, there are no right or wrong answers. We want to hear from everyone in the group. We are pleased you can be part of this group because we think you have important ideas regarding the learning climate in the OR. Don’t hesitate to speak up when you have a point you would like to make.

We will be keeping a record of this discussion so that I don’t have to take notes. I like to follow what is being said and then go back later to review what you said again so I can accurately convey your ideas and opinions.

My role today is to see that we have a productive discussion and to summarize the group’s feelings. I will not refer to any participant by name in the reports. The information will be kept confidential and used only for educational purposes.

1. Questions:

Opening questions

1. What do you like best when working in the OR?

Introductory questions

1. Let’s start the discussion by talking about what makes the OR a good place to learn. What are some of the positive aspects of learning and practicing in the OR?
2. What are some things that aren’t so good about the OR as a place to learn?

Transition questions

1. What was your first impression when you first experienced the educational environment in the OR?

Key questions

1. Which factor effect your learning climate in the OR?
2. Probes for discussion

-Teachers

-Time constrain

-Teamwork and inter-professional collaboration

-Workload and tiredness

-Overall atmosphere in the OR

-Using forms (such as mini-CEX) to evaluate and feedback

Ending questions

1. Of all the things we've talked about, what is most important to you?
2. Is there anything we should have talked about, but didn’t?

Appendix 3

Eliminated items from principle component analysis

|  | Component | | | | | | |
| --- | --- | --- | --- | --- | --- | --- | --- |
| **Eliminated items due to double-factor loading** | 1 | 2 | 3 | 4 | 5 | 6 | 7 |
| 17. My supervisors give regular feedback on my strengths and weaknesses |  |  | .527 |  | .498 |  |  |
| 24. The work I am doing suits my learning objectives at this stage of my training |  | .425 | .413 |  |  |  |  |
| 26. There is enough time in the schedule for me to learn new skills |  | .422 | .414 |  |  |  |  |
| 30. My supervisors treat me as an individual | .622 | .416 |  |  |  |  |  |
| 35. The amount of supervision I receive is appropriate for my level of experience | .580 | .430 |  |  |  |  |  |
| 36. I discuss the management plan of cases with the theatre teacher | .475 | .469 |  |  |  |  |  |
| 37. Whenever I should participate in formal educational programs I get relief from theatre duties | .445 |  |  |  |  |  | -.507 |
| 46. The overall educational environment in the OR is appropriate | .520 | .509 |  |  |  |  |  |
| **Eliminated items due to theoretical irrelevance** | 1 | 2 | 3 | 4 | 5 | 6 | 7 |
| 20. Continuity of care is not affected by differences of opinion between supervisors |  |  |  |  | .553 |  |  |
| 21. Differences of opinion between supervisors about patient management are discussed in such a manner that is instructive to others present |  |  | .400 |  |  |  |  |
| 22. Differences of opinion are not such that they have a negative impact on the work climate |  |  |  |  | .403 |  |  |
| 23. The work I am doing is commensurate with my level of experience |  |  | .487 |  |  |  |  |
| 25. It is possible to do follow up with patients |  |  | .520 |  |  |  |  |

Appendix4 Mean scores and standard deviation of the response from the questionnaires.

| Items | Overall mean scores (±SD) N=140 | SR mean scores (±SD)  N=57 | AR mean scores (±SD)  N=56 | SN mean scores (±SD)  N=27 |
| --- | --- | --- | --- | --- |
| 1. I have good collaboration with theater staff | 3.93 (±0.58) | 3.98 (±0.60) | 3.91 (±0.58) | 3.85 (±0.53) |
| 2. The people I work with are friendly | 3.82 (±0.73) | 3.83 (±0.79) | 3.79 (±0.68) | 3.85 (±0.72) |
| 3. The atmosphere in theatre is pleasant | 3.62 (±0.70) | 3.83 (±0.75) | 3.41 (±0.63) | 3.59 (±0.64) |
| 4. The staff from other discipline dislike it when I practice my skills as the procedure takes longer* | 3.04 (±0.97) | 2.95 (±1.12) | 2.98 (±0.90) | 3.33 (±0.68) |
| 5. I feel discriminated against in theatre because of my sex* | 3.35 (±1.09) | 3.34 (±1.12) | 3.05 (±1.07) | 4.00 (±0.73) |
| 6. I feel part of a team in theatre | 3.70 (±0.78) | 3.78 (±0.88) | 3.55 (±0.76) | 3.81 (±0.56) |
| 7. Attendings, nursing staff, other allied health professionals and residents work together as a team | 3.82 (±0.72) | 3.75 (±0.90) | 3.82 (±0.54) | 4.00 (±0.56) |
| 8. Other allied health professionals make a positive contribution to my training | 3.85 (±0.71) | 3.69 (±0.82) | 3.82 (±0.56) | 4.22 (±0.58) |
| 9. Other allied health professionals are willing to reflect with me on the delivery of patient care | 3.66 (±0.67) | 3.59 (±0.81) | 3.66 (±0.55) | 3.81 (±0.56) |
| 10. Teamwork is an integral part of my training | 4.06 (±0.62) | 4.05 (±0.63) | 4.00 (±0.64) | 4.19 (±0.56) |
| 11. I am asked on a regular basis to provide a rationale for my management decisions and actions | 3.77 (±0.79) | 3.61 (±0.87) | 3.93 (±0.71) | 3.78 (±0.70) |
| 12. My supervisors take the initiative to explain their actions | 3.44 (±0.83) | 3.44 (±0.99) | 3.29 (±0.71) | 3.74 (±0.59) |
| 13. My supervisors take the initiative to evaluate my performance | 3.85 (±0.61) | 3.92 (±0.70) | 3.76 (±0.54) | 3.89 (±0.51) |
| 14. My supervisors take the initiative to evaluate difficult situations I have been involved in | 3.77 (±0.77) | 3.66 (±0.94) | 3.80 (±0.64) | 3.93 (±0.55) |
| 15. My supervisors evaluate whether my performance in patient care is commensurate with my level of training | 3.78 (±0.64) | 3.69 (±0.79) | 3.82 (±0.51) | 3.89 (±0.51) |
| 16. My supervisors assess not only my medical expertise but also other skills such as teamwork, organization or professional behavior | 3.70 (±0.80) | 3.64 (±0.92) | 3.68 (±0.72) | 3.89 (±0.64) |
| 17. My supervisors give regular feedback on my strengths and weaknesses | 3.38 (±0.80) | 3.44 (±0.95) | 3.18 (±0.58) | 3.67 (±0.73) |
| 18. Observation forms (i.e., Mini-CEX) are used to structure feedback | 3.07 (±0.95) | 2.85 (±1.13) | 3.11 (±0.71) | 3.48 (±0.85) |
| 19. Observation forms (i.e., Mini-CEX) are used periodically to monitor my progress | 3.00 (±0.93) | 2.78 (±1.04) | 3.00 (±0.71) | 3.48 (±0.89) |
| 20. Continuity of care is not affected by differences of opinion between supervisors | 3.44 (±0.81) | 3.47 (±0.85) | 3.29 (±0.78) | 3.67 (±0.73) |
| 21. Differences of opinion between supervisors about patient management are discussed in such a manner that is instructive to others present | 3.84 (±0.60) | 3.86 (±0.67) | 3.75 (±0.58) | 4.00 (±0.48) |
| 22. Differences of opinion are not such that they have a negative impact on the work climate | 3.56 (±0.83) | 3.53 (±0.97) | 3.48 (±0.69) | 3.78 (±0.75) |
| 23. The work I am doing is commensurate with my level of experience | 3.73 (±0.56) | 3.74 (±0.61) | 3.64 (±0.52) | 3.89 (±0.51) |
| 24. The work I am doing suits my learning objectives at this stage of my training | 3.76 (±0.63) | 3.67 (±0.76) | 3.79 (±0.53) | 3.93 (±0.47) |
| 25. It is possible to do follow up with patients | 3.88 (±0.59) | 3.93 (±0.59) | 3.77 (±0.66) | 4.00 (±0.39) |
| 26. There is enough time in the schedule for me to learn new skills | 3.48 (±0.81) | 3.35 (±0.92) | 3.43 (±0.71) | 3.85 (±0.66) |
| 27. My supervisors take time to explain things when asked for advice | 3.69 (±0.68) | 3.63 (±0.75) | 3.64 (±0.70) | 3.93 (±0.39) |
| 28. My supervisors are happy to discuss patient care | 3.77 (±0.65) | 3.65 (±0.77) | 3.82 (±0.58) | 3.93 (±0.47) |
| 29. There is (are) NO supervisor(s) who have a negative impact on the educational climate | 3.04 (±0.93) | 3.11 (±0.96) | 2.86 (±0.84) | 3.30 (±0.99) |
| 30. My supervisors treat me as an individual | 3.64 (±0.67) | 3.50 (±0.74) | 3.73 (±0.65) | 3.74 (±0.53) |
| 31. My supervisors treat me with respect | 3.59 (±0.74) | 3.54 (±0.83) | 3.55 (±0.74) | 3.78 (±0.51) |
| 32. My supervisors are all in their own way positive role models | 3.75 (±0.68) | 3.79 (±0.77) | 3.71 (±0.62) | 3.74 (±0.59) |
| 33. When I need a supervisor, I can always contact one | 3.75 (±0.73) | 3.67 (±0.79) | 3.77 (±0.74) | 3.89 (±0.58) |
| 34. When I need to consult a supervisor, they are readily available | 3.76 (±0.67) | 3.72 (±0.77) | 3.77 (±0.60) | 3.85 (±0.60) |
| 35. The amount of supervision I receive is appropriate for my level of experience | 3.74 (±0.65) | 3.68 (±0.78) | 3.73 (±0.56) | 3.85 (±0.53) |
| 36. I discuss the management plan of cases with the theatre teacher | 3.61 (±0.70) | 3.51 (±0.83) | 3.59 (±0.60) | 3.89 (±0.51) |
| 37. Whenever I should participate in formal educational  programmes I get relief from theatre duties | 3.69 (±0.96) | 3.68 (±1.06) | 3.73 (±0.88) | 3.63 (±0.93) |
| 38. I am often too tired to get the most out of theatre teaching* | 2.87 (±0.94) | 2.98 (±1.16) | 2.86 (±0.75) | 2.67 (±0.78) |
| 39. I am so stressed in theatre that I do not learn as much as I could* | 3.13 (±0.89) | 3.36 (±0.97) | 3.16 (±0.71) | 2.56 (±0.80) |
| 40. I am clear about the learning objectives of the theatre teaching session | 3.72 (±0.61) | 3.74 (±0.66) | 3.61 (±0.59) | 3.93 (±0.47) |
| 41. There is a clinical training programme here that allows me to  get first-hand experience in a range of procedures | 3.57 (±0.86) | 3.21 (±1.06) | 3.75 (±0.64) | 3.96 (±0.44) |
| 42. I feel responsible and accountable for the care given to my patients | 3.88 (±0.62) | 3.83 (±0.73) | 3.88 (±0.54) | 4.00 (±0.48) |
| 43. I am aware of my professional role in the theatre | 3.91 (±0.61) | 3.97 (±0.62) | 3.80 (±0.64) | 4.00 (±0.48) |
| 44. I have an appropriate level of clinical responsibility | 3.87 (±0.61) | 3.83 (±0.68) | 3.89 (±0.56) | 3.93 (±0.55) |
| 45. I have the opportunity to acquire the appropriate practical  procedures for my level of training | 3.82 (±0.68) | 3.72 (±0.83) | 3.86 (±0.55) | 3.96 (±0.52) |
| 46. The overall educational environment in the OR is appropriate | 3.63 (±0.70) | 3.59 (±0.82) | 3.59 (±0.63) | 3.81 (±0.56) |
